# Ononin combined with *Lactobacillus paracasei* alleviates DSS-induced ulcerative colitis by ferroptosis via inhibiting JAK2/STAT3 signaling pathway

**DOI:** 10.1016/j.bbrep.2026.102482

**Published:** 2026-02-05

**Authors:** Mei Huang, Feng Yang, Ju-cai Song, Xue Wang, Pan-pan Jiao, Meng-jie Kang, Qian-qian Guo, Liang-zhu Si, Shu-han Zhang, Lin-shan Luo, Yong-wei Li, Wei Zhang, Yue-sheng Gong, Lei Dong

**Affiliations:** a Henan University of Chinese Medicine, Zhengzhou, 450002, China; b Zhengzhou Anorectal Hospital Affiliated of Hospital of Henan University of Chinese Medicine, Zhengzhou, 450004, China; c Department of Medical Laboratory, Henan Provincial Hospital of Traditional Chinese Medicine, Dongfeng Road, Zhengzhou, 450002, China

**Keywords:** Ulcerative colitis, Ononin, *Lactobacillus paracasei*, Ferroptosis, JAK2/STAT3 pathway

## Abstract

**Background:**

Ulcerative colitis (UC) is a chronic inflammatory bowel disease with a complex pathogenesis and limited treatment options. This study aimed to evaluate the therapeutic effect of Ononin combined with *Lactobacillus paracasei* in a dextran sulfate sodium (DSS)-induced murine UC model and to elucidate the underlying mechanisms involving ferroptosis and the JAK2/STAT3 signaling pathway.

**Methods:**

A UC model was established by administering 3% DSS in drinking water to male BALB/c mice. Therapeutic efficacy was assessed based on changes in body weight, disease activity index (DAI), colon length, and histopathological alterations. Serum levels of inflammatory cytokines (TNF-α, IL-1β) and oxidative stress markers (MPO, MDA, SOD, GSH) were quantified. Protein expression related to ferroptosis (ACSL4, GPX4), intestinal barrier integrity (Occludin, Claudin-1), and the JAK2/STAT3 pathway was analyzed in colon tissue.

**Results:**

Combined treatment with Ononin and *Lactobacillus paracasei* significantly alleviated UC symptoms, as shown by improved body weight, colon length, DAI scores, and histopathology. It also reduced pro-inflammatory cytokine levels, suppressed oxidative stress and neutrophil infiltration, and enhanced antioxidant capacity. Mechanistically, the treatment downregulated ACSL4 expression, inhibited JAK2/STAT3 phosphorylation, and upregulated the expression of GPX4, Occludin, and Claudin-1.

**Conclusion:**

The combination of Ononin and *Lactobacillus paracasei* effectively ameliorated DSS-induced colitis in mice. Its protective mechanism involves suppressing inflammation and oxidative stress, inhibiting ferroptosis (potentially through blocking the JAK2/STAT3 pathway), and enhancing intestinal barrier integrity. These findings provide a strong preclinical rationale for developing this combination as a potential therapeutic strategy for UC.

## Highlights

• The combination of Ononin and *L. paracasei* synergistically ameliorates DSS-induced ulcerative colitis.
• The combinatory therapy inhibits the JAK2/STAT3 signaling pathway in colonic tissue.
• The protective effect is achieved by triggering ferroptosis in pathogenic colon cells.
• We first link JAK2/STAT3 inhibition to ferroptosis in UC treatment.
• Provides a novel “natural product-probiotic” therapeutic strategy for UC.

## 1 Introduction

Ulcerative colitis (UC) is a chronic and frequently relapsing nonspecific inflammatory disease of the intestinal tract. Its typical symptoms include periodic diarrhea, abdominal pain, and the passage of mucus, pus, and blood in the stools [1]. In severe cases, complications such as intestinal perforation, hemorrhage, and colorectal carcinoma may occur [2]. At present, the exact etiology and pathogenesis of UC have not been fully elucidated. It is generally accepted that dysbiosis of the intestinal microbiota, compromised intestinal mucosal barrier function, and abnormal immune regulation significantly contribute to the pathogenesis of UC [3]. Among these factors, the impairment of the intestinal mucosal barrier is regarded asa key mechanism underlying the development of UC [4]. In clinical practice, several treatment options exist for UC, though most primarily focus on symptom management. Currently, drug therapy remains the predominant approach, utilizing medications such as aminosalicylates, corticosteroids, and immunosuppressants [5]. However, conventional drug therapies are often associated with significant side effects that can severely impact patients' quality of life. Therefore, exploring novel therapeutic strategies that are safe, effective, and have minimal side effects is essential for UC treatment.

Ferroptosis is a novel form of regulated cell death characterized by iron-dependent lipid peroxidation, which distinguishes it from other forms of cell death such as apoptosis, necrosis, pyroptosis, and autophagy [6,7]. Recent studies suggest that ferroptosis may play a critical role in the pathogenesis of UC, offering a potential new target for therapeutic intervention. For instance, sodium butyrate (NaB) has been shown to alleviate inflammatory bowel disease (IBD) by inhibiting ferroptosis and modulating the ERK/STAT3 signaling pathway and intestinal flora [8]. Furthermore, Zhu et al. observed that in DSS-induced colitis, upregulation of HMGB1 coincided with altered TLR4/NF-κB/GPX4 expression and elevated ferroptosis-related genes. They also found that HMGB1 inhibition alleviated inflammation, restored intestinal barrier function, and suppressed ferroptosis [9]. Notably, ferroptosis inhibitors have demonstrated the potential to ameliorate colitis pathology in preclinical models, suggesting that targeting this pathway may represent a promising therapeutic strategy for ulcerative colitis in the future.

In recent years, natural flavonoids have gained increasing attention as potential modulators of ferroptosis, owing largely to their notable antioxidant and metal-chelating properties. A growing body of evidence suggests that several flavonoids can influence disease progression through ferroptosis regulation. For example, quercetin has been shown to alleviate kainic acid–induced cognitive impairment via the Nrf2-dependent ferroptosis pathway, while baicalein promotes ferroptosis in colorectal cancer cells by inhibiting the JAK2/STAT3 pathway, leading to the suppression of GPX4 transcription [10,11]. Furthermore, chrysin—another naturally occurring flavonoid—has been reported to confer protection against cerebral ischemia–reperfusion injury by inhibiting ferroptosis through the HIF-1α/CP loop, and it also mitigates UC by reducing inflammation and modulating gut microbiota composition [12,13]. Notably, the ferroptosis-inhibitory effect of chrysin has also been demonstrated in the context of acute lung injury, where chrysin-loaded PLGA nanoparticles were shown to attenuate ferroptosis by upregulating Nrf2-dependent antioxidant responses [14]. These findings underscore the context-dependent roles of flavonoids in ferroptosis and their therapeutic potential across diverse diseases.

Ononin, a flavonoid compound derived from widely used traditional Chinese medicinal herbs such as *Astragalus membranaceus* and *Pueraria lobata*, has been demonstrated by modern pharmacological studies to possess antioxidant, antitumor, and anti-inflammatory properties [15]. Some studies have shown that Ononin can inhibit the expression of inflammatory factors, reduce intestinal inflammatory responses, regulate the composition of intestinal microbiota, promote the growth of beneficial bacteria, and exhibit potential therapeutic effects on intestinal diseases such as UC [16,17]. *Lactobacillus paracasei*, a probiotic species belonging to the *Lactobacillus* genus, is commonly found in both traditional fermented dairy products and the human gastrointestinal tract [18]. It exhibits notable biological activities, including antioxidant and anti-inflammatory properties, and has the capacity to enhance the intestinal environment, restore microbial balance, repair the intestinal mucosal barrier, and reduce intestinal inflammation [19]. Furthermore, *Lactobacillus paracasei* shows significant therapeutic potential for a variety of intestinal diseases, with UC being one of the most prominent [20].

However, the specific role of Ononin in modulating ferroptosis in the context of UC, and whether its combination with *Lactobacillus paracasei* may exert a synergistic and enhanced therapeutic effect through targeting this pathway, remain unexplored. Therefore, this study employed a dextran sulfate sodium (DSS)-induced murine model of UC to investigate the combined therapeutic effects of Ononin and *Lactobacillus paracasei*, with particular emphasis on elucidating their roles in regulating ferroptosis. Through this investigation, we aim to evaluate the synergistic potential of this combination and clarify the underlying mechanisms, thereby providing new insights for the clinical management of UC.

### 1.1 Animals

Fifty-four male specific pathogen-free (SPF) BALB/c mice, aged 6–8 weeks with body weights of 20–22 g, were supplied by Liaoning Changsheng Biotechnology Co., Ltd. All animals were housed in a standard SPF environment maintained at 22 ± 2 °C and 55 ± 5% relative humidity, under a 12-h light/dark cycle. Mice had free access to sterilized tap water and a standard commercial diet. All experimental procedures were conducted in accordance with the Guidelines for the Humane Use of Animals issued by the National Institutes of Health and were approved by the Animal Research Welfare Committee of Henan University of Traditional Chinese Medicine Affiliated Zhengzhou Anorectal Hospital (Ethics Approval No. ZG20250302A).

## 2 Materials and methods

### 2.1 Materials

Dextran sulfate sodium (DSS; molecular weight: 36,000–50,000 Da) was purchased from Yea Sen Biotechnology (Shanghai, China; Catalogue No. 60316ES76). Ononin was obtained from Yuan Ye Bio-Technology (Shanghai, China; Catalogue No. B20214). Commercial ELISA kits for myeloperoxidase (MPO), malondialdehyde (MDA), superoxide dismutase (SOD), glutathione (GSH), tumor necrosis factor-α (TNF-α), interleukin-1β (IL-1β) were acquired from Jiangsu Enzyme Exemption Industry Co., Ltd (Jiangsu, China; Catalogue Nos. MM-0338M1, MM-0897M1, MM-0389M1, MM-0661M1, MM-0132M1, and MM-0040M1 respectively). *Lactobacillus paracasei* was sourced from BNCC (Hebei, China; Strain ID: BNCC337289). Mesalazine sustained-release granules were obtained from Shanghai Ethypharm Pharmaceuticals Co., Ltd (Shanghai, China; Catalogue No. 230826). All primary and secondary antibodies were purchased from BioDragon Technology Co., Ltd. (Jiangsu, China), including: acyl-CoA synthetase long-chain family member 4 (ACSL4, Catalogue No. RM8089), glutathione peroxidase 4 (GPX4, Catalogue No. RM8391), Occludin (10Z10) rabbit monoclonal antibody (Catalogue No. RM4965), Claudin-1 rabbit polyclonal antibody (Catalogue No. BD-PT0942), Janus kinase 2 (JAK2) rabbit polyclonal antibody (Catalogue No. RM4299), phospho-JAK2 (Y1007/Y1008; clone 17F11) rabbit monoclonal antibody (Catalogue No. RM4972), signal transducer and activator of transcription 3 (STAT3) rabbit monoclonal antibody (Catalogue No. RM4186), phospho-STAT3 rabbit monoclonal antibody (Catalogue No. RM4968), β-actin mouse monoclonal antibody (Catalogue No. B1029), HRP-conjugated goat anti-rabbit IgG (H + L) (Catalogue No. BF03008X), and HRP-conjugated goat anti-mouse IgG (H + L) (Catalogue No. BF03001X).

### 2.2 Methods

#### 2.2.1 Preparation of bacterial suspension

*Lactobacillus paracasei* strains were inoculated into conical flasks containing MRS liquid medium and cultured in a constant-temperature shaker at 37 °C for 18 h. After incubation, bacterial cultures were harvested by centrifugation, resuspended, and adjusted to a concentration of 3.33 × 10^9^ CFU/mL for subsequent use.

#### 2.2.2 Model construction and drug intervention

During the acclimatization period, mice were randomly assigned to groups using a random number table (n = 9/group) after 7 days: a control group (CON), a DSS-induced colitis group (DSS) [21], a mesalazine group (5-ASA, 200 mg/kg) [22], an Ononin group (Ononin, 20 mg/kg) [17], a *Lactobacillus paracasei* group (LP, 3.33 × 10^9^ CFU/mL) [23], and a combination treatment group(Ononin + LP). Following acclimatization, the DSS, 5-ASA, Ononin, LP, and Ononin + LP groups received 3% DSS in drinking water ad libitum for 7 consecutive days to induce UC, whereas the CON group received double-distilled water. Typically, after 5–7 days of DSS exposure, the mice developed characteristic symptoms of colitis, including perianal soiling, mucopurulent bloody stools, and diarrhea. The successful induction of the model was preliminarily confirmed by a significant elevation in the disease activity index (DAI) in three randomly selected mice. After this confirmation, a 7-day oral gavage treatment (10 mL/kg/day) was initiated. Each treatment group received its respective intervention at the indicated doses; the CON and DSS groups received an equal volume of normal saline. Twenty-four hours after the final administration, mice were deeply anesthetized with sodium pentobarbital (50 mg/kg, i.p.) and subsequently euthanized. Blood and colon tissues were collected for further analysis. The overall experimental timeline is illustrated in the Supplementary File.

#### 2.2.3 Disease activity index (DAI) score

After the successful establishment of the DSS-induced colitis model, all mice were weighed daily at the same time. Observations were recorded for general condition (including mental state and coat appearance), food and water intake, stool consistency, and the presence of hematochezia. The disease activity index (DAI) was calculated for each animal based on the scoring criteria shown in Table 1, using the following formula: DAI = (weight loss score + stool consistency score + fecal bleeding score)/3. The evaluation was performed as previously described [24].

**Table 1:** Quantitative table of Disease Activity Index (DAI) score.

Weight loss (%)	Feces consistency	Hemafecia	Score
0	Normal	N/A	0
1–5	Mild soft	Slight bleeding	1
5–10	Soft and wet	Moderate bleeding	2
10–20	Half loose stool	Gross bleeding	3
>20	loose stool	Blood clot around anus	4

#### 2.2.4 Determination of colon length

The colon was carefully excised, and its morphology was observed and documented. Colon length was subsequently measured.

#### 2.2.5 Measurement of spleen index

The spleens of mice were isolated and weighed. The spleen index was calculated as follows: (spleen weight/body weight) × 100%.

#### 2.2.6 Hematoxylin-eosin staining and histopathological assessment

Colon tissue samples were fixed in 4% paraformaldehyde, embedded in paraffin, and sectioned. Sections were deparaffinized, rehydrated through a graded ethanol series, and stained with hematoxylin and eosin (H&E). After staining, slides were dehydrated, cleared in xylene, and mounted for microscopic examination. Histopathological evaluation was performed under a light microscope. Histological scoring was performed independently by two investigators blinded to the treatment groups. The severity of colonic inflammation was assessed using a histopathological damage score adapted from a previous study [25], as detailed below: 0, normal; 1, mild inflammation with <10% crypt loss and focal enterocyte hyperplasia; 2, moderate inflammation with 10%–30% crypt loss, multifocal enterocyte hyperplasia, and goblet cell loss; 3, severe inflammation with 30%–50% crypt loss, diffuse enterocyte hyperplasia, and few goblet cells; 4, extensive inflammation with >50% crypt loss, diffuse enterocyte hyperplasia, and mucosal ulceration.

#### 2.2.7 Detection of inflammatory factors TNF-α and L-1β content

Serum samples collected from mice were analyzed for levels of TNF-α, and IL-1β using commercially available ELISA kits according to the manufacturer's instructions.

#### 2.2.8 Measurement of oxidative stress markers

Serum samples were collected from mice, and the levels of MPO, MDA, SOD, and GSH were measured using commercially available ELISA kits according to the manufacturer's instructions.

#### 2.2.9 Detection of protein expression level

Colon tissue samples were collected from mice, and proteins were extracted using a commercial tissue protein extraction kit according to the manufacturer's instructions. Protein concentrations were determined using the bicinchoninic acid (BCA) assay. Protein lysates were then denatured by heating at high temperature for 10 min. Subsequent steps included electrophoresis, membrane transfer, blocking, and overnight incubation at 4 °C with the following primary antibodies: ACSL4, GPX4, JAK2, p-JAK2, STAT3, p-STAT3, Occludin, and Claudin-1. After washing with Tris-buffered saline containing Tween 20 (TBST), membranes were incubated with horseradish peroxidase (HRP)-conjugated secondary antibodies (1:10,000 dilution) for 1 h at room temperature. Protein bands were visualized using enhanced chemiluminescence (ECL) and imaged. Band intensities were quantified with ImageJ software, using β-actin as the loading control. Relative protein expression levels were calculated as the ratio of the target protein band intensity to that of β-actin. All experiments were performed in triplicate.

#### 2.2.10 Statistical analysis

All data were statistically analyzed using GraphPad Prism 8.0.2 and are expressed as mean ± standard deviation (SD). Each experiment was performed in at least three independent replicates. Comparisons among multiple groups were conducted using one-way analysis of variance (ANOVA), followed by Duncan's multiple range test, while comparisons between two groups were assessed using Student's t-test. A *p*-value <0.05 was considered statistically significant.

## 3 Results

### 3.1 Ononin combined with *Lactobacillus paracasei* ameliorated the symptoms of DSS-induced colitis mice

DSS-induced colitis is characterized by marked body weight loss, diarrhea, and severe bloody stools. In this study, the DAI score of the CON group remained stable at 0 throughout the experiment, and the mice exhibited healthy growth. In contrast, mice in the DSS group showed significant body weight loss compared with the normal group (*p* < 0.05, Fig. 1B). After drug intervention, weight loss improved in all treatment groups, accompanied by a significant reduction in DAI scores (*p* < 0.01, Fig. 1C). The combined treatment group demonstrated more pronounced body weight recovery and a greater decrease in DAI scores compared to other groups (*p* < 0.001). These results suggest that Ononin combined with *Lactobacillus paracasei* can effectively alleviate colitis symptoms in UC mice.

**Fig. 1:**
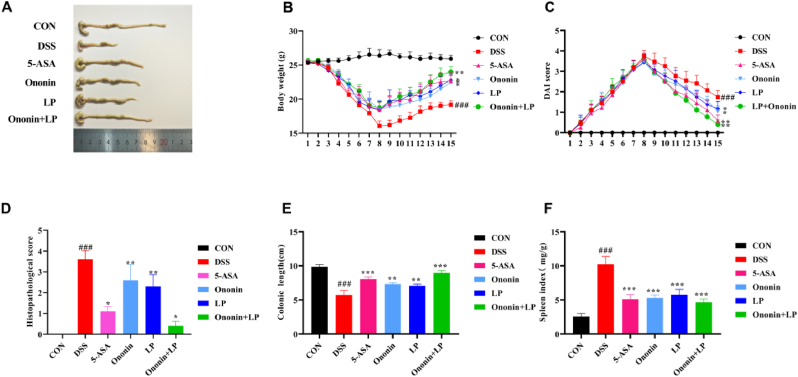
Effects of Ononin combined with *Lactobacillus paracasei* on ameliorating the general symptoms of murine colitis. (A) Representative image of colon lengths. (B) Changes in body weight of mice following the induction of colitis. (C) Daily disease activity index (DAI) of mice after colitis induction. (D) Histopathological score. (E) Colon length. (F) Spleen index. All data are presented as mean ± standard deviation. ^###^*p* < 0.001 vs. CON group, ∗*p* < 0.05, ∗∗*p* < 0.01, ∗∗∗*p* < 0.001 vs. DSS group.

### 3.2 Comparison of colon length in mice in each group

Compared to the CON group, DSS administration led to significant colon shortening, a clear indicator of colonic inflammation (*p* < 0.001, Fig. 1A). Following drug treatment, colon shortening was markedly alleviated relative to the DSS group (*p* < 0.001). Among all treatment groups, the combination of Ononin and *Lactobacillus paracasei* (Ononin + LP) most effectively suppressed colon shortening, showing highly significant improvement (*p* < 0.001, Fig. 1A and E). In contrast, no statistically significant differences were observed in colon length among the Ononin, *Lactobacillus paracasei*, and 5-ASA monotherapy groups (*p* > 0.05).

### 3.3 Comparison of spleen index of mice in each group

The spleen, a major lymphoid organ, enlarges in response to systemic infection or inflammation. In this study, compared with the CON group, mice in the DSS group exhibited a significantly higher splenic index (*p* < 0.001, Fig. 1F). This suggests that UC mice not only suffer from intestinal damage but also develop spleen injury. Following drug intervention, the splenic index decreased significantly in all treatment groups relative to the DSS group (*p* < 0.01). Notably, the reduction in the splenic index was most pronounced in the Ononin + *Lactobacillus paracasei* (Ononin + LP) group (*p* < 0.001, Fig. 1F), indicating that this combination can effectively ameliorate DSS-induced systemic inflammation and spleen damage in UC mice.

### 3.4 Comparison of histopathology of the colon of mice in each group

As shown in Fig. 2, mice in the CON group exhibited intact intestinal mucosa and glands, with well-organized morphology and structure. No inflammatory cell infiltration, ulcer formation, or obvious submucosal edema was observed. In contrast, the DSS group displayed severe damage to intestinal wall integrity, including marked intestinal wall thickening, significant reduction of glands, destruction of crypt architecture, extensive inflammatory cell infiltration, and widespread ulceration, indicating a more severe degree of pathological injury in colonic tissues. Following drug intervention, all treatment groups showed significant improvement in colonic histology. The area of inflammatory cell infiltration decreased, glandular and crypt structures became more orderly and organized, and submucosal edema was alleviated. Among these groups, the Ononin + LP group exhibited the most notable pathological recovery, with repaired colonic mucosal epithelium, absence of obvious edema or wall thickening, and substantially reduced inflammatory cell infiltration.

**Fig. 2:**
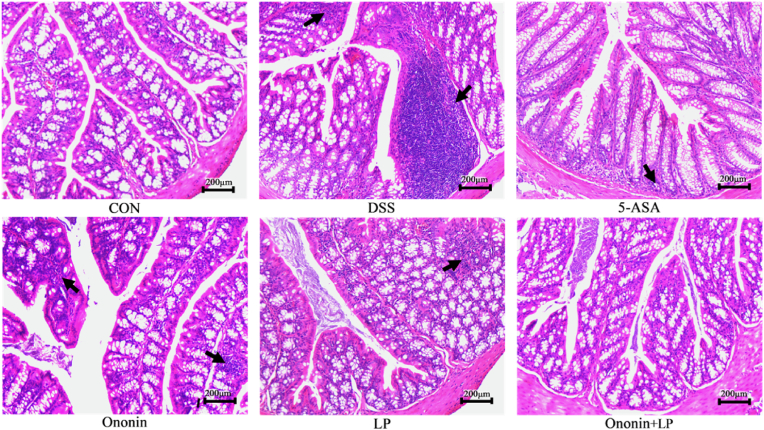
Histopathological analyses of H&E-stained colon tissue sections in a DSS-induced Ulcerative colitis (UC) model in mice. Arrows indicated the inflammatory infiltration, mucosal erosion, and damage of crypts. Scale bar = 200 μm.

Consistent with histological observations, the histopathological score was significantly higher in the DSS group than in the CON group (*p* < 0.001, Fig. 1D). This increase was significantly attenuated by treatment with Ononin, LP, or their combination (*p* < 0.01 or *p* < 0.05 vs. DSS group, Fig. 1D), with the most pronounced reduction observed in the Ononin + LP group. These results indicate that Ononin combined with *Lactobacillus paracasei* alleviates DSS-induced colitis in mice and represents a promising candidate for the treatment of UC.

### 3.5 Comparison of serum levels of inflammatory factors TNF-α, and IL-1β in mice in each group

Compared with the CON group, the DSS group showed significantly elevated serum levels of TNF-α and IL-1β (*p* < 0.01, Fig. 3A and B), indicating enhanced colonic inflammation. Following drug intervention, serum levels of these inflammatory cytokines were significantly reduced in all treatment groups—including the 5-ASA, Ononin, *Lactobacillus paracasei*, and Ononin + LP groups—compared with the DSS group (*p* < 0.01 or *p* < 0.001). Among them, the Ononin + LP group and the 5-ASA group exhibited the most pronounced decrease (*p* < 0.001, Fig. 3A and B). These results suggest that the protective effect of Ononin combined with *Lactobacillus paracasei* against UC is associated with its ability to modulate inflammatory responses.

**Fig. 3:**
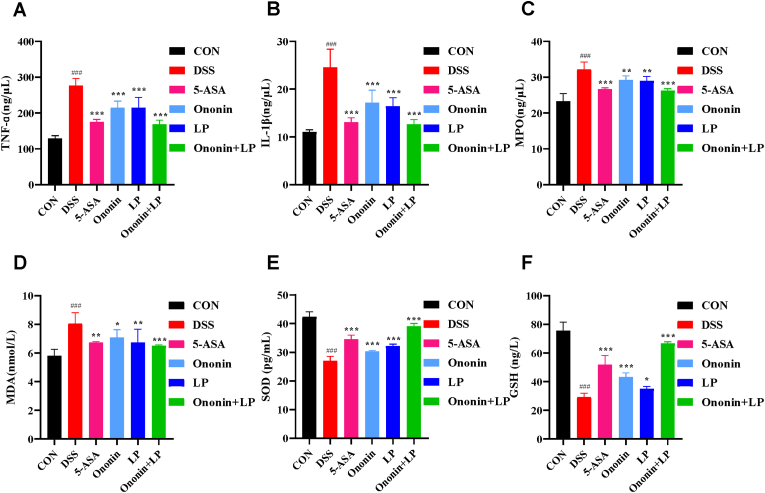
Effects of Ononin combined with *Lactobacillus paracasei* on pro-inflammatory factors and oxidative stress levels in mice. (A) Serum tumor necrosis factor-α (TNF-α) levels. (B) Serum interleukin-1β (IL-1β) levels. (C) Serum myeloperoxidase (MPO) levels. (D) Serum malondialdehyde (MDA) levels. (E) Serum superoxide dismutase (SOD) levels. (F) Serum glutathione (GSH) levels. All data are presented as mean ± standard deviation. ^###^*p* < 0.001 vs. CON group; ∗*p* < 0.05, ∗∗*p* < 0.01, ∗∗∗*p* < 0.001 vs. DSS group.

### 3.6 Comparison of serum levels of MPO, MDA, SOD and GSH in mice in each group

Compared with the CON group, mice in the DSS group exhibited significantly elevated serum levels of MPO and MDA (*p* < 0.01, Fig. 3C and D), along with markedly reduced levels of SOD and GSH (*p* < 0.01, Fig. 3E and F), indicating impaired free-radical scavenging and enhanced oxidative stress. Following drug intervention, serum levels of MPO and MDA were significantly lower in the 5-ASA, Ononin, *Lactobacillus paracasei*, and Ononin + LP groups compared with the DSS group (*p* < 0.01 or *p* < 0.001, Fig. 3C and D), while levels of SOD and GSH were significantly increased (*p* < 0.05 or *p* < 0.01, Fig. 3E and F). Among all treatment groups, the Ononin + LP group showed the most pronounced reduction in MPO and MDA (*p* < 0.001), suggesting a notable recovery in free-radical elimination capacity. These results indicate that Ononin combined with *Lactobacillus paracasei* can effectively enhance systemic antioxidant defenses and thereby alleviate UC.

### 3.7 Comparison of the relative expression levels of Occludin and Claudin-1 proteins in the colonic tissues of mice in each group

Compared with the CON group, the DSS group demonstrated significantly lower protein expression of Occludin and Claudin-1 in colonic tissues (*p* < 0.001, Fig. 4A, B, 4C). All drug-treated groups exhibited a significant reversal of this decrease, showing elevated expression of both proteins relative to the DSS group (*p* < 0.01). Notably, the Ononin + LP and 5-ASA groups displayed the most pronounced upregulation (*p* < 0.001, Fig. 4A, B, 4C). These findings indicate that the combination of Ononin and *Lactobacillus paracasei* effectively ameliorates DSS-induced disruption of the intestinal mucosal barrier.

**Fig. 4:**
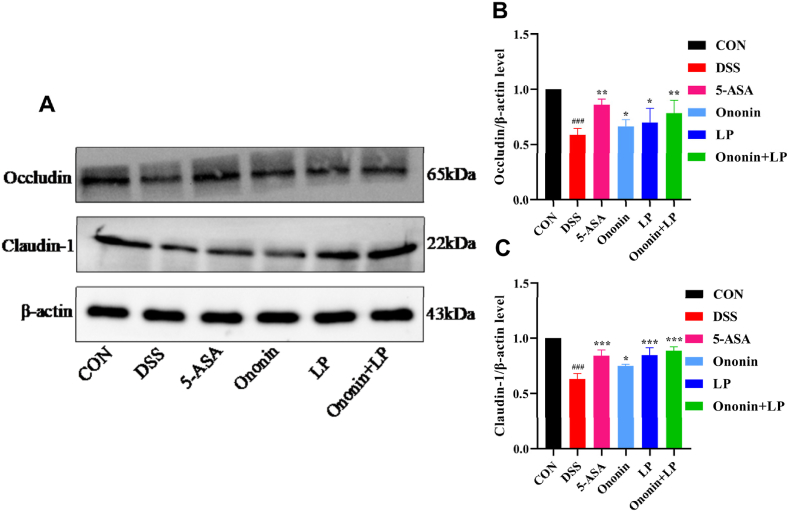
Effect of Ononin combined with *Lactobacillus paracasei* on the colon tissue protein levels of Occludin and Claudin-1 were examined by Western blot, (A) Representative western blotting images of Occludin and Claudin-1 in each group.(B) Occludin. (C) Claudin-1. Relative levels of proteins were qualified by ImageJ software and was normalized by corresponding total protein content. All data were presented as mean ± standard deviation. ^###^*p* < 0.001, vs CON group; ∗*p* < 0.05,∗∗*p* < 0.01,∗∗∗*p* < 0.001, vs DSS group.

### 3.8 Comparison of differences in the protein expression levels of ACSL4 and GPX4 in the colonic tissues of mice in each group

Compared with the CON group, mice in the DSS group exhibited significantly increased expression of ACSL4 protein and significantly decreased expression of GPX4 (*p* < 0.01, Fig. 5A, B, 5C). Following drug intervention, all treatment groups showed significantly reduced ACSL4 levels and elevated GPX4 expression relative to the DSS group (*p* < 0.01, Fig. 5A, B, 5C). Among these groups, the Ononin + LP group displayed the most pronounced alterations in both proteins (*p* < 0.001). These results suggest that the combination of Ononin and *Lactobacillus paracasei* effectively attenuates ferroptosis in colonic tissue.

**Fig. 5:**
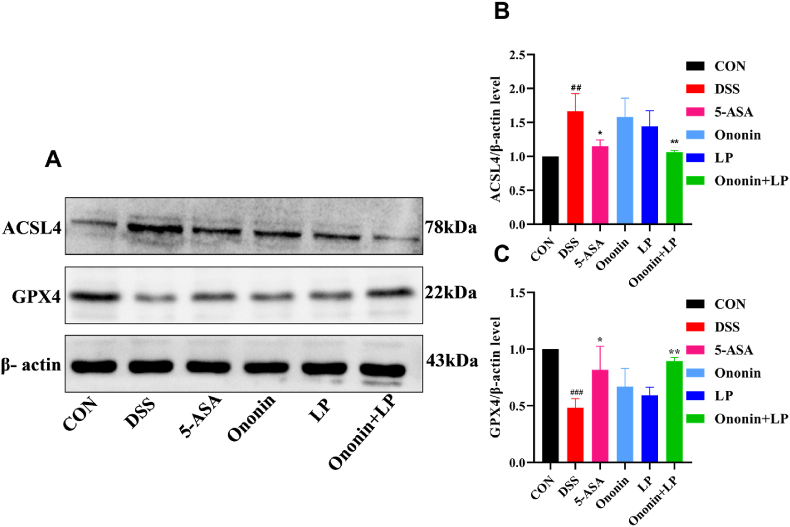
Effect of Ononin combined with *Lactobacillus paracasei* on the colon tissue protein levels of acyl-CoA synthetase long-chain family member 4 (ACSL4)and glutathione peroxidase 4 (GPX4) were examined by Western blot, (A) Representative western blotting images of ACSL4 and GPX4 in each group. (B) ACSL4. (C) GPX4. Relative levels of proteins were qualified by ImageJ software and was normalized by corresponding total protein content. All data were presented as mean ± standard deviation. ^###^*p* < 0.001, vs CON group; ∗*p* < 0.05,∗∗*p* < 0.01,∗∗∗*p* < 0.001, vs DSS group.

### 3.9 Comparison of the differences in the expression levels of p-JAK2 and p-STAT3 proteins in the colonic tissues of mice in each group

Compared with the CON group, mice in the DSS group showed significantly increased expression levels of p-JAK2 and p-STAT3 proteins in colonic tissues (*p* < 0.01, Fig. 6A, B, 6C). Following drug intervention, all treatment groups exhibited significantly decreased expression of these proteins relative to the DSS group (*p* < 0.01, Fig. 6A, B, 6C). Among them, the Ononin + LP group displayed the most pronounced reduction in p-JAK2 and p-STAT3 expression compared to the other groups (*p* < 0.001). These results indicate that the combination of Ononin and *Lactobacillus paracasei* suppresses JAK2/STAT3 phosphorylation, thereby attenuating inflammation and ferroptosis, which collectively contribute to anti-inflammatory and cytoprotective outcomes.

**Fig. 6:**
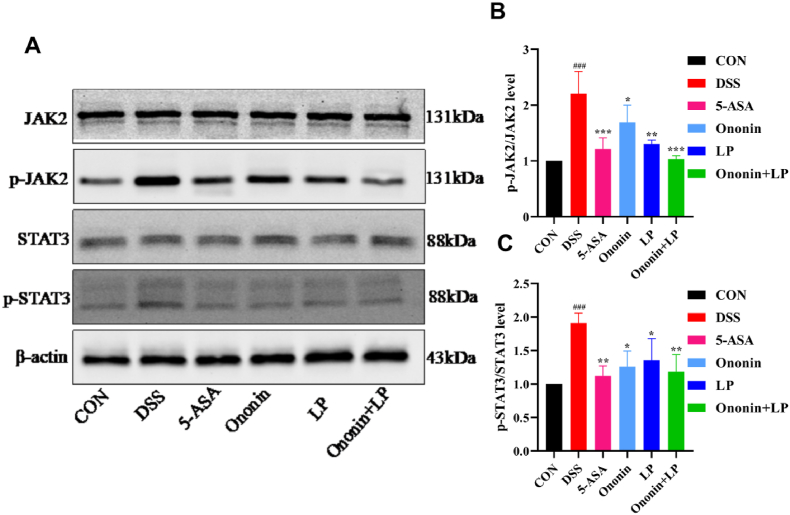
The effect of Ononin combined with *Lactobacillus paracasei* on the Janus kinase 2 (JAK2)/signal transducer and activator of transcription 3 (STAT3) signaling pathway in a DSS-induced UC model in mice is presented. (A) Representative Western blot images showing the levels of phospho-JAK2 (p-JAK2), JAK2, phospho-STAT3 (p-STAT3), and STAT3 in each group. (B) The ratio of p-JAK2 to JAK2. (C) The ratio of p-STAT3 to STAT3. Relative phosphorylation levels of the proteins were quantified using ImageJ software and normalized to the corresponding total protein content. All data are presented as mean ± standard deviation. ^###^*p* < 0.001 vs. CON group; ∗*p* < 0.05, ∗∗*p* < 0.01, ∗∗∗*p* < 0.001 vs. DSS group.

## 4 Discussion

Based on its clinical manifestations, UC is categorized in Traditional Chinese Medicine (TCM) under syndromes such as “diarrhea,” “dysentery,” “intestinal wind,” “chronic dysentery,” “intestinal pi,” and “hematochezia.” In recent years, TCM has demonstrated distinct advantages in treating UC, with numerous studies showing that it achieves significant clinical efficacy through multi-component, multi-target, and multi-mechanism approaches [26]. Astragalus (Huang Qi), a widely used traditional Chinese herb, is recognized for its effectiveness in enhancing immune function and reducing inflammation [27]. Ononin, a key bioactive component derived from Astragalus, effectively downregulates the overexpression of IL-1β and TNF-α while suppressing abnormal activation of the MAPK and NF-κB pathways. This dual action inhibits the IL-1β-induced pro-inflammatory response in chondrocytes and prevents degradation of the extracellular matrix, thereby ameliorating inflammatory conditions [16]. These findings highlight the therapeutic potential of Ononin in inflammatory diseases. Furthermore, studies indicate that Ononin alleviates DSS-induced colitis by promoting mitophagy and inhibiting the NLRP3 inflammasome [17]. In gut microbiota research, *Lactobacillus paracasei* has been shown to mitigate intestinal inflammation and promote gut health, effectively preventing or treating diseases related to gut dysbiosis [28]. Specifically, *Lactobacillus paracasei* NTU 101 alleviates DSS-induced UC by enhancing antioxidant enzymes (GR, GSH, CAT, SOD) and reducing MDA and IFN-γ levels [19]. Previous studies have confirmed the individual therapeutic effects of Ononin and *Lactobacillus paracasei* in DSS-induced UC. Our current study further demonstrates that the combination of Ononin and *Lactobacillus paracasei* as an adjunctive therapy exhibits superior efficacy compared to Ononin alone in the treatment of UC.

Intestinal inflammation is the central pathogenic mechanism in the active phase of UC, characterized by persistent infiltration of inflammatory cells in the mucosa—a hallmark pathological feature throughout the course of the disease [29]. TNF-α and IL-1β are key pro-inflammatory cytokines, primarily secreted by monocytes and macrophages, and their levels are markedly elevated during active UC [30]. TNF-α acts as a critical signaling molecule in immune regulation and the initiation of inflammatory responses [31]. IL-1β, produced by activated mononuclear macrophages, plays a pivotal role in acute and chronic inflammation as well as in autoimmune processes; its concentration often reflects the severity of UC [32].In this study, serum levels of TNF-α and IL-1β were significantly increased in DSS-induced UC mice compared to the CON group. After drug intervention, both cytokine levels were reduced relative to the DSS group. These findings suggest that the combination therapy may alleviate intestinal immune inflammation and promote mucosal repair by more effectively downregulating serum TNF-α and IL-1β in UC mice.

Oxidative stress refers to a pathological process characterized by an imbalance between reactive oxygen species (ROS) and antioxidants in vivo, which can lead to cellular and tissue damage [33]. Lipid peroxidation is recognized as a significant contributing factor to UC. By impairing intestinal mucosal barrier function, triggering inflammatory responses, disrupting mucosal immunity, and hindering mucosal repair, oxidative stress plays a central role in disease pathogenesi [34]. Consequently, targeting oxidative stress has emerged as a promising therapeutic strategy for UC. This approach is supported by interventions such as *Amauroderma rugosum* extract, which alleviates UC by regulating macrophage polarization and suppressing oxidative stress, and vanillic acid, which attenuates ROS accumulation in immune cells [[34], [35], [36]]. Furthermore, recent mechanistic studies have revealed that proteins such as AMBRA1 can exacerbate oxidative stress by regulating key redox sensors like NRF2, highlighting the complexity of this therapeutic target [37]. The assessment of oxidative stress in both experimental and clinical contexts relies on specific biomarkers. In UC, aberrant oxidative stress promotes the release of MPO, leading to overactivation of intestinal immune cells and exacerbation of intestinal inflammation [38]. SOD, an essential antioxidant enzyme, plays a vital role in neutralizing excess free radicals and mitigating oxidative cellular damage [39]. MDA, the terminal product of ROS-induced lipid peroxidation, serves as a key biomarker for evaluating cellular damage, with its concentration directly reflecting the extent of oxidative injury [40]. GSH, a critical intracellular non-enzymatic antioxidant, is crucial for defense against oxidative stress and participates in regulating cell proliferation, apoptosis, and immune function [41]. It has been widely used as a biomarker for inflammation and oxidative stress, indicating the severity of colonic mucosal damage [42]. Therefore, measuring serum levels of MPO, MDA, SOD, and GSH in UC mice allows for the assessment of lipid peroxidation extent, thereby enabling evaluation of intestinal mucosal damage and oxidative stress levels associated with the condition. The results of this study demonstrate that the combination of Ononin and *Lactobacillus paracasei* enhances SOD and GSH activity, reduces MPO and MDA levels, and effectively inhibits oxidative stress responses.

Studies have confirmed that intestinal epithelial dysfunction is a critical factor in the onset and recurrence of UC, with tight junctions (TJs) serving as key determinants of normal intestinal epithelial barrier function [43]. TJs are composed of structural proteins such as Occludin, Claudin-1, Zonula occludens (ZO) proteins, and junctional adhesion molecules (JAM) [44]. Both Occludin and Claudin-1 act as biomarkers for intestinal epithelial barrier integrity. Under physiological conditions, these proteins form continuous, chain-like structures that bridge adjacent epithelial cells, thereby regulating epithelial permeability [45]. Consequently, modulating TJs to maintain epithelial barrier integrity represents a crucial therapeutic strategy for UC. The results of this study demonstrated that, compared to the CON group, mice in the DSS group exhibited significantly downregulated expression of Occludin and Claudin-1 proteins in colonic tissues. Following treatment with Ononin and *Lactobacillus paracasei*, the inflammatory state was significantly alleviated, and the expression of Occludin and Claudin-1 proteins was significantly upregulated. These findings suggest that the pathogenesis of UC may be closely associated with the abnormal expression of proteins integral to the intestinal mechanical barrier.

Ferroptosis is a novel form of programmed cell death characterized by iron-dependent lipid peroxidation and the accumulation of ROS [46]. Growing evidence indicates that the pathogenesis of UC is closely associated with intestinal iron overload and dysregulated iron metabolism. A diet rich in polyunsaturated fatty acids (PUFAs) exacerbates colitis by promoting ferroptosis; the damage signal HMGB1 induces ferroptosis in intestinal cells via the TLR4/NF-κB/GPX4 pathway, thereby disrupting the epithelial barrier; moreover, Coix seed oil (CSO) alleviates experimental UC by simultaneously protecting the intestinal barrier, inhibiting ferroptosis, and modulating the gut microbiota [[47], [48], [49]]. These findings collectively establish ferroptosis as a key pathological mechanism and a promising therapeutic target in UC.

Notably, ACSL4 and GPX4 are recognized as critical regulators of ferroptosis. GPX4 functions as the central enzyme that catalyzes the reduction of lipid peroxides in cells, thereby preventing cytotoxicity and subsequent ferroptosis [50]. Conversely, ACSL4 serves as an essential biomarker of ferroptosis; its upregulation not only promotes lipid peroxide production but also significantly suppresses GPX4 expression, ultimately leading to impaired ROS clearance, oxidative stress, and the induction of ferroptosis [7,51]. In this study, we observed that DSS-induced UC mice exhibited decreased GPX4 expression and increased ACSL4 expression in intestinal mucosal tissues, indicating the occurrence of ferroptosis. These results collectively suggest that the therapeutic effects of Ononin combined with *Lactobacillus paracasei* in alleviating intestinal mucosal damage in UC mice are mediated, at least in part, through the inhibition of ferroptosis pathways.

The JAK2/STAT3 signaling pathway is a critical mediator of oxidative stress and inflammatory activation [52]. Its sustained activation in intestinal epithelial cells initiates a pathological cascade: phosphorylated JAK2 activates STAT3, which then translocates to the nucleus and promotes the transcription of pro-inflammatory and pro-ferroptotic genes [53]. Recent studies indicate that persistent JAK2/STAT3 activation further promotes ferroptosis in intestinal epithelial cells by enhancing lipid peroxidation and depleting glutathione [54]. The therapeutic potential of targeting this axis is supported by natural compounds such as Baicalin and Biochanin A, which alleviate colitis through inhibition of JAK2/STAT3 and its downstream inflammatory and ferroptotic cascades [54,55]. These findings collectively establish JAK2/STAT3 as a promising multi-target pathway for UC intervention.

Building on this established framework, our study provides novel mechanistic insights. We demonstrate that the combination of Ononin and *Lactobacillus paracasei* confers significant protection against DSS-induced colitis. Mechanistically, this protective effect is associated with modulation of the JAK2/STAT3 pathway. Specifically, Ononin and *Lactobacillus paracasei* treatment effectively suppressed the phosphorylation of JAK2 and STAT3, thereby disrupting downstream signal transduction. This inhibition correlated with a marked reduction in inflammatory factor production and attenuated ferroptosis in intestinal epithelial cells, as reflected by the restored expression of key ferroptosis markers. Collectively, our findings indicate that the Ononin and LP combination ameliorates intestinal mucosal injury, at least in part, by inhibiting the JAK2/STAT3 signaling pathway and its associated ferroptotic cascade, thereby interrupting the vicious cycle of inflammation and epithelial cell death in UC.

In summary, our study demonstrates that the combination of Ononin and *Lactobacillus paracasei* effectively attenuates DSS-induced UC in mice. This protective effect is associated with the inhibition of colonic inflammation and ferroptosis. Mechanistically, the Ononin and *Lactobacillus paracasei* treatment suppressed the JAK2/STAT3 signaling pathway, downregulated pro-inflammatory cytokines (TNF-α and IL-1β), modulated oxidative stress markers (MPO, SOD, MDA, GSH), and restored the expression of tight-junction proteins (Occludin, Claudin-1) and ferroptosis-related regulators (ACSL4, GPX4).

Collectively, our findings elucidate the mechanism through which Ononin together with *Lactobacillus paracasei* ameliorates UC and may contribute to the development of novel therapeutic strategies for this disease.

### 4.1 Limitations of the study

The present study has several limitations that should be considered. First, the sample size was relatively small, which may affect the generalizability of the findings. Second, the experimental model used was an acute UC model; therefore, the therapeutic effect of Ononin combined with *Lactobacillus paracasei* in chronic UC remains unclear and warrants further investigation. Third, while this study preliminarily explored the role of oxidative stress, ferroptosis, and the JAK2/STAT3 signaling pathway, the precise mechanistic interactions among these pathways under the combined regimen require further elucidation. Additionally, although we evaluated key local molecular mechanisms in colon tissue (e.g., barrier function and signaling pathways), the assessment of oxidative stress and inflammatory cytokines was performed in serum. These systemic measurements reflect the overall bodily burden and therapeutic response but may not fully capture the specific concentrations or dynamics within the colonic mucosa. Future studies should aim to include larger sample sizes, employ chronic UC models, and incorporate direct quantification of oxidative and inflammatory markers in colon tissue homogenates to provide a more detailed picture of the local milieu. Investigating the combination of Ononin with other probiotic strains to assess potential synergistic effects also represents a valuable direction for further research.

## Clinical trial number

Not applicable.

## Funding

This work was supported by the Special Project of Traditional Chinese Medicine Science Research in Henan Province (2023ZY1027, 2024ZY3107, 2025ZY3115) and China Medical Education Association 2024 Medical Science and Technology Research Project (2024KTM019).

## CRediT authorship contribution statement

**Mei Huang:** Conceptualization, Data curation, Formal analysis, Investigation, Methodology, Visualization, Writing – original draft, Writing – review & editing. **Feng Yang:** Conceptualization, Data curation, Formal analysis, Investigation, Methodology, Writing – review & editing. **Ju-cai Song:** Conceptualization, Writing – review & editing. **Xue Wang:** Formal analysis, Investigation, Methodology. **Pan-pan Jiao:** Investigation. **Meng-jie Kang:** Investigation. **Qian-qian Guo:** Investigation. **Liang-zhu Si:** Investigation. **Shu-han Zhang:** Investigation. **Lin-shan Luo:** Formal analysis, Resources, Software, Supervision. **Yong-wei Li:** Visualization, Writing – review & editing. **Wei Zhang:** Conceptualization, Writing – review & editing. **Yue-sheng Gong:** Conceptualization, Funding acquisition, Project administration, Writing – review & editing. **Lei Dong:** Conceptualization, Resources, Software, Visualization, Writing – review & editing.

## Declaration of competing interest

The authors declare no competing financial interests.

## Figures and Tables

**figs1:**
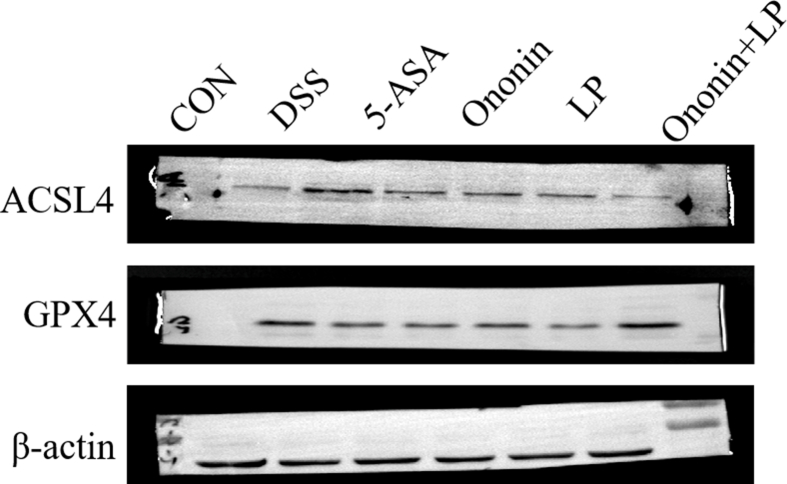


**figs2:**
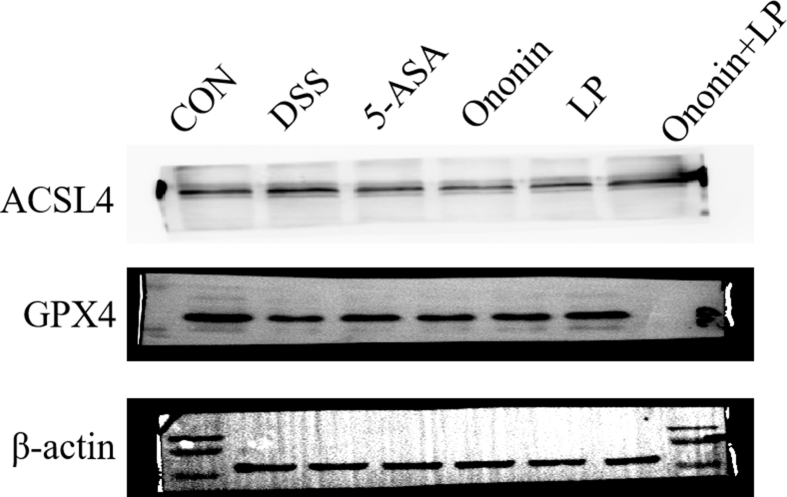


**figs3:**
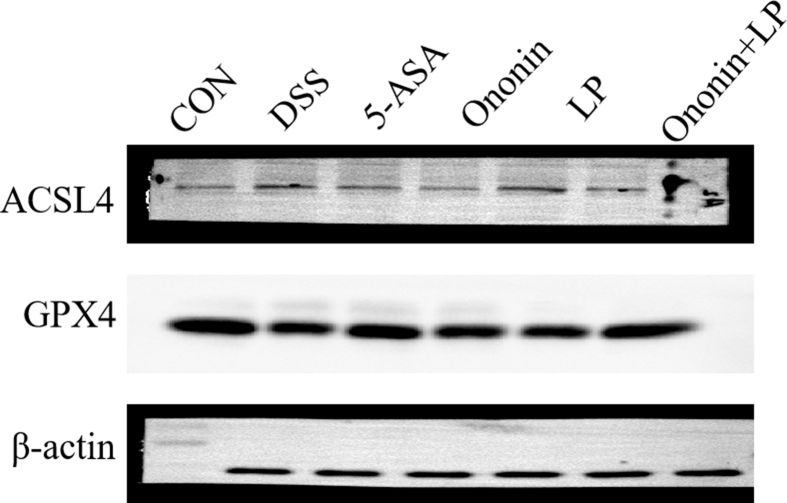


**figs4:**
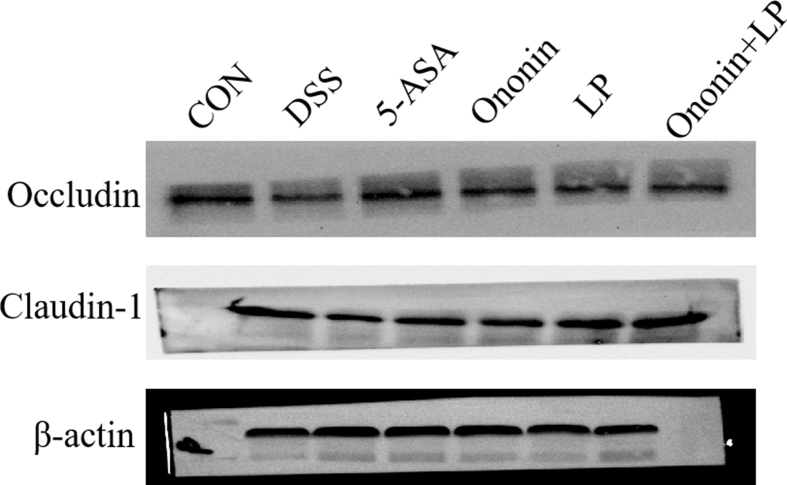


**figs5:**
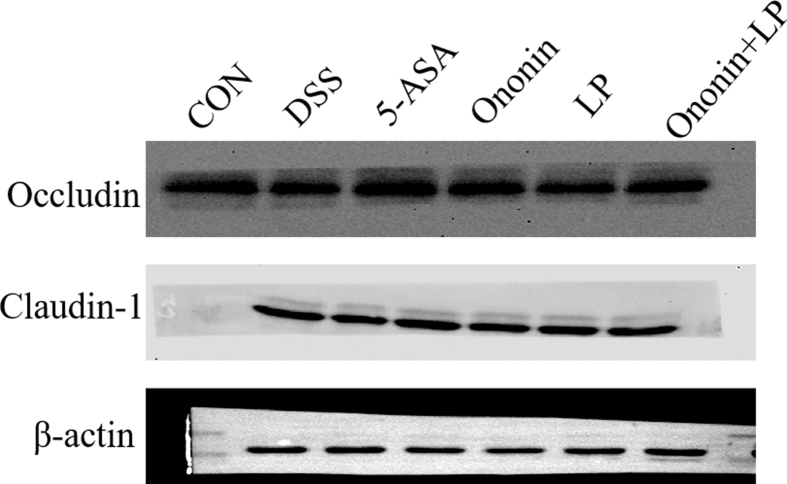


**figs6:**
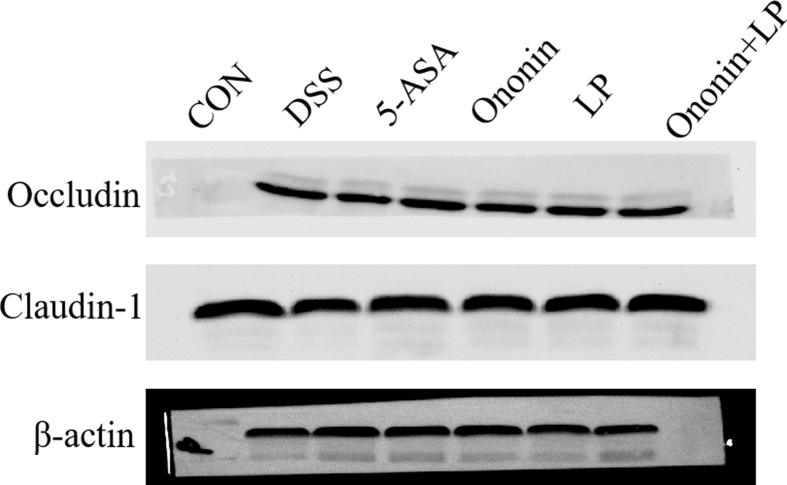


**figs7:**
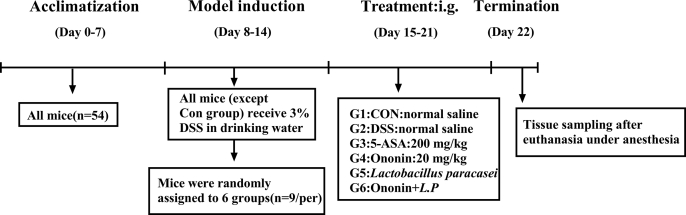


**figs8:**
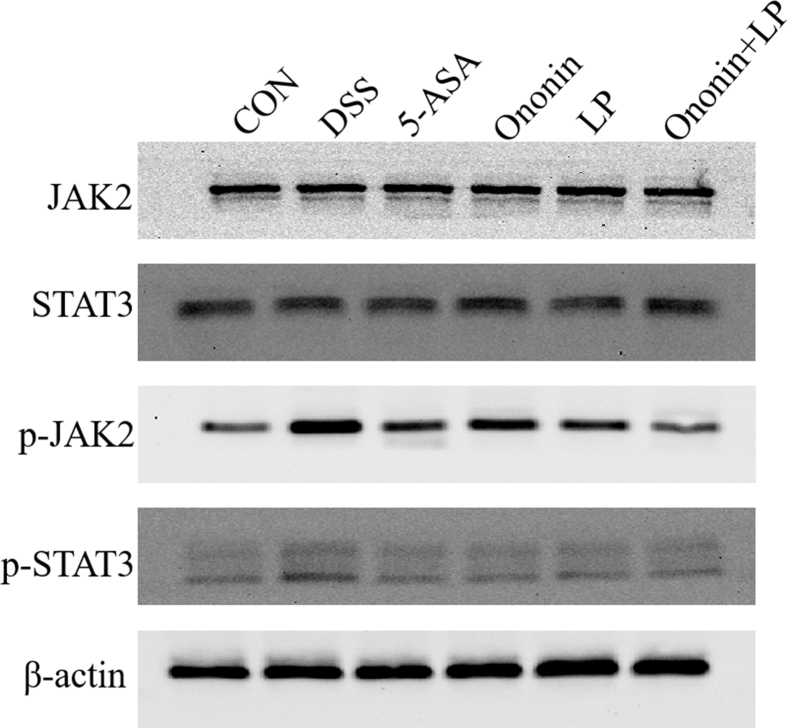


**figs9:**
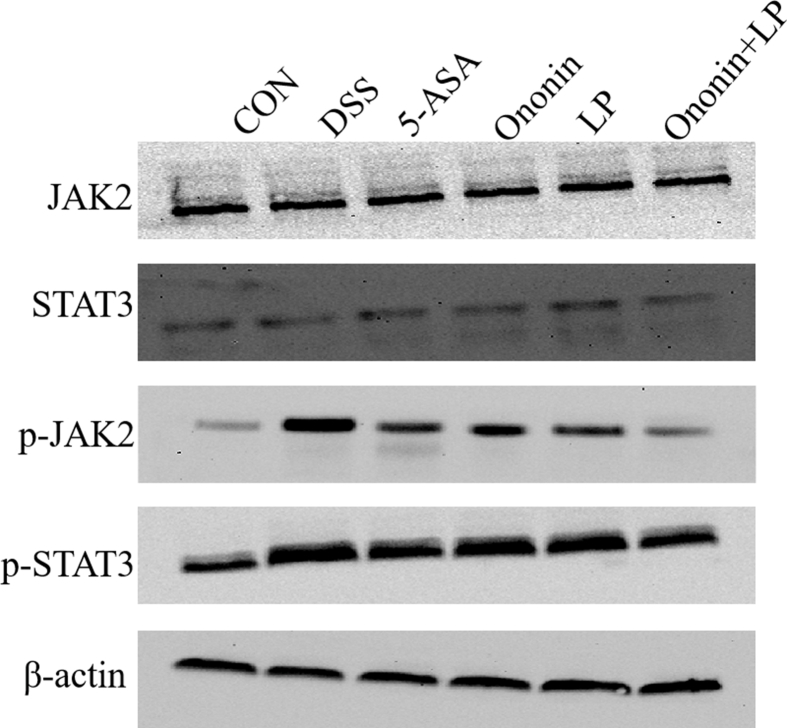


**figs10:**
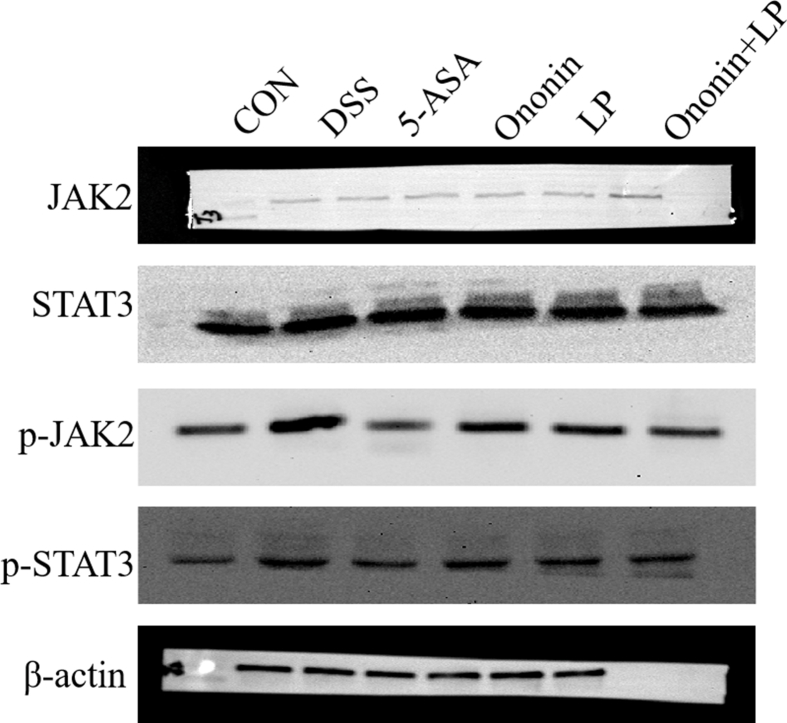


## Data Availability

Data will be made available on request.
